# Profiling the effect of nafcillin on HA-MRSA D712 using bacteriological and physiological media

**DOI:** 10.1038/s41597-019-0331-z

**Published:** 2019-12-17

**Authors:** Akanksha Rajput, Saugat Poudel, Hannah Tsunemoto, Michael Meehan, Richard Szubin, Connor A. Olson, Anne Lamsa, Yara Seif, Nicholas Dillon, Alison Vrbanac, Joseph Sugie, Samira Dahesh, Jonathan M. Monk, Pieter C. Dorrestein, Rob Knight, Victor Nizet, Bernhard O. Palsson, Adam M. Feist, Joe Pogliano

**Affiliations:** 1 Department of Bioengineering, University of California, San Diego, La Jolla USA; 2 Division of Biological Sciences, University of California San Diego, La Jolla, CA 92093 USA; 3 Collaborative Mass Spectrometry Innovation Center, University of California, San Diego, La Jolla, California USA; 4 Skaggs School of Pharmacy and Pharmaceutical Sciences, University of California San Diego, La Jolla, CA USA; 5 Department of Pediatrics, University of California, San Diego, La Jolla, CA USA; 6 Collaborative to Halt Antibiotic-Resistant Microbes (CHARM), Department of Pediatrics, UC San Diego, La Jolla, CA 92093 USA; 7 Center for Marine Biotechnology and Biomedicine, Scripps Institution of Oceanography, University of California San Diego, La Jolla, CA 92093 United States of America; 8 Center for Microbiome Innovation, University of California San Diego, La Jolla, CA 92093 USA; 9 Department of Computer Science and Engineering, University of California San Diego, La Jolla, CA 92093 USA; 10 Novo Nordisk Foundation Center for Biosustainability, Technical University of Denmark, Kemitorvet, Building 220, 2800 Kongens, Lyngby, Denmark

**Keywords:** Clinical microbiology, Bacteriology, Antimicrobial resistance

## Abstract

*Staphylococcus aureus* strains have been continuously evolving resistance to numerous classes of antibiotics including methicillin, vancomycin, daptomycin and linezolid, compounding the enormous healthcare and economic burden of the pathogen. Cation-adjusted Mueller-Hinton broth (CA-MHB) is the standard bacteriological media for measuring antibiotic susceptibility in the clinical lab, but the use of media that more closely mimic the physiological state of the patient, e.g. mammalian tissue culture media, can in certain circumstances reveal antibiotic activities that may be more predictive of effectiveness *in vivo*. In the current study, we use both types of media to explore antibiotic resistance phenomena in hospital-acquired USA100 lineage methicillin-resistant, vancomycin-intermediate *Staphylococcus aureus* (MRSA/VISA) strain D712 *via* multidimensional high throughput analysis of growth rates, bacterial cytological profiling, RNA sequencing, and exo-metabolomics (HPLC and LC-MS). Here, we share data generated from these assays to shed light on the antibiotic resistance behavior of MRSA/VISA D712 in both bacteriological and physiological media.

## Abstract (summary)

**Table:** 

Measurement(s)	Antibacterial Response • cDNA • transcription profiling assay • culture medium • organic acid
Technology Type(s)	bacterial cytological profiling • RNA sequencing • liquid chromatography-tandem mass spectrometry • high-performance liquid chromatography
Factor Type(s)	growth medium
Sample Characteristic - Organism	Staphylococcus aureus

Machine-accessible metadata file describing the reported data: 10.6084/m9.figshare.10283108

## Background & Summary

*Staphylococcus aureus* is among the major contributors of nosocomial infection worldwide. The pathogen is responsible for various life-threatening diseases like pneumonia, sepsis, toxic shock syndrome, and endocarditis^1^. Over the course of time, *S*. *aureus* has sequentially developed resistance to various clinical antibiotics including penicillin, methicillin, aminoglycosides, fluoroquinolones and many more^2^. An alarming recent problem is potential resistance to vancomycin, which emerged in association with hospitalization, prolonged infection, and extended vancomycin treatment. Commonly, methicillin-resistant *Staphylococcus aureus* (MRSA) are treated with vancomycin, which can predispose to stepwise increases in resistance progressing from vancomycin-intermediate (VISA) strains and ultimately strains became completely resistant to vancomycin (VRSA). VISA is thought to be epigenetic and triggered by prolonged exposure to even non-glycopeptide classes of antibiotics like beta-lactams^2,3^, and currently VISA strains are much more frequent in the clinical setting than VRSA strains. In the present study, we focus on hospital acquired USA100 lineage VISA strain D712, previously collected from a patient in late phase of vancomycin and daptomycin treatment^4^.

Cation-adjusted Mueller-Hinton broth (CA-MHB) is the standard media used to test the quantitative susceptibility of antimicrobial agents in clinical microbiology laboratories around the world. CA-MHB contains low thymidine and thymine while enriched in calcium and magnesium salts, and allows reproducible measurement of minimal inhibitory concentration (MIC) against diverse clinically-relevant bacterial species^5^. Recent research has shown that the media more closely mimic physiological conditions in the human patient, for example the common mammalian tissue culture media Roswell Park Memorial Institute 1640 (1640) might reveal useful antibiotic activities missed in CA-MHB^6,7^.

In the present study, we focused on elucidating the effect of nafcillin on VISA strain D712 in both bacteriological (CA-MHB) and physiological media (RPMI supplemented with 10% Luria broth to achieve grow equivalence). Between these two media the MIC value decreased from 256 µg/ml in CA-MHB to 1 µg/ml in RPMI + 10%LB. The effect of nafcillin at various sub-inhibitory concentrations were monitored using growth curves, bacterial cytological profiling (BCP), RNA sequencing, and exo-metabolomics (HPLC and LC-MS) high throughput experiments. The BCP assay was done to identify cytological parameters that change in response to antibiotic treatment RNAseq and metabolomics were used to measure changes in gene expression and secreted metabolites in response to the presence of nafcillin.

## Methods

The methods are adapted from our previously published paper^8^.

### Culture and growth conditions

**Fig. 1:**
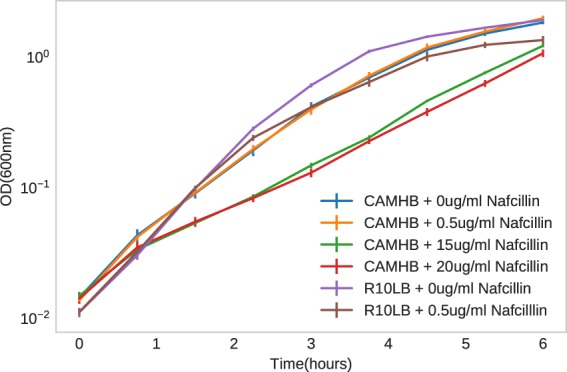
Growth curve for *Staphylococcus aureus* D712 strain in presence of nafcillin at various sub-inhibitory concentrations in CA-MHB and R10LB media.

Standard bacteriological media MHB (Sigma-Aldrich) was supplemented with 25 mg/L Ca^2+^ and 12.5 mg/L Mg2+ (CA-MHB). Eukaryotic cell culture media Roswell Park Memorial Institute 1640 (RPMI) (Thermo Fisher Scientific) was supplemented with 10% LB (R10LB). Broth microdilution was performed to determine the nafcillin MIC in each media condition. On the day of the experiment, overnight cultures of HA-MRSA D712 were diluted to a starting OD600 of 0.01 into fresh media and grown at 37 °C with stirring to OD600 0.4. This preculture was then diluted back to OD600 0.01 into fresh media containing no drug or sub-inhibitory concentrations of nafcillin relative for each media type. Growth was monitored by obtaining OD600 readings every 45 min for 6 hr. Three biological replicates were collected for the study, each derived from different colony and overnight culture. The growth curve is provided as Fig. 1.

### Bacterial cytological profiling

At the 3 hour mark, samples were taken for fluorescence microscopy, similar to previously described with modifications^9–11^. In brief, 8 µL cells were added to 2 µL dye mix containing 10 µg/mL DAPI, 2.5 µM SYTOX Green, and 60 µg/mL FM4-64 in 1x T-base. The sample was then transferred to a glass slide containing an agarose pad (20% media, 1.2% agarose) and imaged on an Applied Precision DV Elite epifluorescence microscope with a CMOS camera. The exposure times for each wavelength were as follows, TRITC/Cy-5 = 0.025 s, FITC/FITC = 0.01 s, DAPI/DAPI = 0.015 s, and were kept constant for all images.

**Fig. 2:**
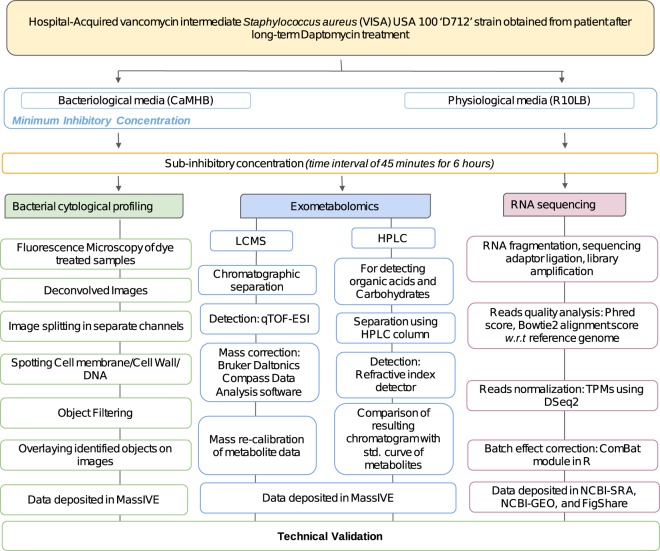
Diagram depicting the methodology of high throughput approaches used to profile the *Staphylococcus aureus* D712 in presence of nafcillin.

Deconvolved images were adjusted using FIJI (ImageJ 1.51w) and Adobe Photoshop (2015.1) to remove background in WGA and DAPI channels and to ensure that cell and DNA objects are within the highest intensity quartile. These images were then processed using a custom CellProfiler 3.0 pipeline that individually threshold and filtered WGA and DAPI channels to obtain segmentation masks for the cell wall, DNA and entire cell. Objects identified in this manner were further processed in CellProfiler to obtain a total of 5285 features^12,13^. Prior to analysis, feature selection is necessary to create a subset of relevant features as to minimize redundancy within the dataset. The summary of processing steps is presented in Fig. 2.

### cDNA library preparation and RNA sequencing

After 3 hours of growth, 3 mL samples were taken for RNA sequencing and added to tubes containing 6 mL RNAprotect. After incubation, they were centrifuged to remove the supernatant. RNA was extracted from the pelleted cells using a ‘Quick RNA Fungal/Bacterial Microprep’ kit developed by Zymo Research. The cells were mechanically lysed with a Roche MagNa Lyser instrument and DNA was removed with DNase I during the RNA purification. RNA quality was checked with an Agilent Bioanalyzer instrument and ribosomal RNA was removed using an Illumina Ribo-Zero kit. The remaining RNA was used to build a cDNA library for sequencing using a KAPA Stranded RNA-seq Library Preparation Kit. The kit was used for RNA fragmentation, sequencing adapter ligation, and library amplification. The generated cDNA libraries were sent for Illumina sequencing on a HiSeq. 4000.

### RNA sequencing analysis

The reference genome *S*. *aureus* D712 was submitted in NCBI with accession number VFJD01000001.1. The genome coverage of reference genome S. aureus D712 is 60X with final genome size is 2,825,989 bp. D712 is an evolved strain of D592. Both D592 and D712 strains were collected from the same patient upon pre-daptomycin treatment and after daptomycin treatment, respectively.

The phred quality scores for illumina sequencing were generated using Fastqc package^14^. Bowtie2 was used to align the raw reads to D712 genome^15^ and to calculate alignment percentage, FastQC^16^. The aligned reads were then normalized to transcripts per million (TPM) with DESeq. 2. The ComBat module within the sva package of R was used to correct for batch effects^17,18^. Distance matrix for hierarchical clustering were calculated with sklearn package^19^. The summary steps are provided in Fig. 2.

### Untargeted liquid chromatography mass spectrometry data acquisition

Following dilution of the preculture of HA-MRSA USA100 D712 into fresh media, approximately 400 µL of liquid media containing cells were collected at 45 minutes intervals (at the same time as samples for OD600 measurements) from each of the samples. Growth media was syringe-filtered through 0.22 µm disc filters (Millex-GV, MilliporeSigma) to remove cells. The filtered growth media was collected and stored at −80 °C until analysis by liquid chromatography mass spectrometry (LC/MS). For LC/MS analysis, samples were subjected to chromatographic separation using an UltiMate 3000 UHPLC system (Thermo Scientific). Chromatographic separations were achieved using a 50 mm × 2.1 mm Kinetex 2.6 micron polar-C18 column (Phenomenex) held at a fixed temperature of 30 °C within an actively heated column compartment. Samples were injected onto the LC column via thermostatted autosampler maintained at 4 °C. For samples containing RPMI + 10% LB media the injection volume was 5 µl, while the injection volume was 2 µl for samples containing CA-MHB to prevent excessive oversaturation of the mass spectrometer detector due to the higher concentrations of many molecules in the CA-MHB media.

After injection, the sample components were eluted from the LC column into the mass spectrometer using a flow rate of 0.5 mL/min and the following mobile phases: Mobile phase A was LC/MS grade water with 0.1% formic acid (v/v) and mobile phase B was LC/MS grade acetonitrile with 0.1% formic acid (v/v). The LC gradient program was as follows: 0–1.0 min 5%B, 1.0–5.0 min 5–35%B, 5.0–5.5 min 35–100%B, 5.5–6.0 min 100%B, and 6.0–6.5 min 100–5%B followed by 5 minutes of re-equilibration at 5%B. Mass spectrometric data was acquired using a Bruker Daltonics maXis Impact quadrupole-time-of-flight (qTOF) mass spectrometer equipped with an Apollo II electrospray ionization (ESI) source and controlled via otofControl v4.0.15 and Hystar v3.2 software packages (Bruker Daltonics). The mass accuracy of the maXis instrument was first externally calibrated using a calibration solution of sodium formate which provided >21 reference m/z’s between 50–1500 m/z of the mass spectrum in both positive and negative polarities (reference m/z list provided within instrument control software). Sodium formate solution was prepared using 9.9 ml of 50/50% isopropanol/water, plus 0.2% formic acid, and 100 μl of 1 M NaOH. During infusion of all samples, the mass accuracy of the instrument was maintained to <10 ppm via constant introduction of and internal calibrant, or “lock mass”, in the form of hexakis (1 H,1 H,2H-difluoroethoxy)phosphazene (SynQuest Labs, Inc.). During positive polarity runs the lock mass compound was detected as the ion m/z 622.028960 (C12H19F12N3O6P3+) and in negative polarity the lock mass compound formed the ion m/z 556.001951 (C10H15F10N3O6P3−).

Instrument source parameters were set as follows: nebulizer gas (Nitrogen) pressure, 2 Bar; Capillary voltage, 3,500 V; ion source temperature, 200 °C; dry gas flow, 9 L/min. The global mass spectral acquisition rate was set at 3 Hz. The instrument transfer optics were tuned as follows: Ion funnel 1 & 2 RFs of 250 Vpp (volts peak-to-peak), hexapole RF of 100 Vpp, quadrupole ion energy of 5 eV, collision quadrupole energy of 5 eV, and a TOF pre-pulse storage of 7.0 µsecs. The post-collision quadrupole RF and TOF transfer time were stepped across four values per MS scan. The collision RF was stepped at 450, 550, 800, 1100 Vpp. The transfer time was stepped at 70, 75, 90, 95 µsecs. All samples were run twice, once under positive polarity settings and once under negative polarity settings.

Following acquisition of the LC/MS data, lock mass calibration was applied to all data files in order to apply a linear correction calibration to all m/z values recorded in each mass spectrum. The application of this mass correction was applied automatically via the Bruker Daltonics Compass Data Analysis software (ver. 4.3.110), using the m/z of the hexakis (1 H,1 H,2H-difluoroethoxy) phosphazene as the reference lock mass calibration compound. Following lock mass re-calibration of the data, all files were converted from the Bruker Daltonics proprietary format (.d) and exported to an open data format known as.mzXML. All data herein was deposited to MassIVE^20^. The brief methodology is provided in Fig. 2.

### Targeted high-performance liquid chromatography

For organic acid and carbohydrate detection, samples were collected every 45 minutes and filtered as described above. The filtered samples were loaded onto a 1260 Infinity series (Agilent Technologies) high-performance liquid chromatography (HPLC) system with an Aminex HPX-87H column (Bio-Rad Laboratories) and a refractive index detector. The system was operated using ChemStation software. The HPLC was run with a single mobile phase composed of HPLC grade water buffered with 5 mM sulfuric acid (H2SO4). The flow rate was held at 0.5 mL/minute, the sample injection volume was 10 uL, and the column temperature was maintained at 45 °C. The identities of compounds were determined by retention time comparison to standard curves of acetate, ethanol, glucose, lactate, pyruvate, and succinate. The peak area integration and resulting chromatograms were generated within ChemStation and compared to that of the standard curves in order to determine the concentration of each compound in the samples. These final concentration values were deposited to MassIVE database^20^. The procedure of HPLC is depicted in Fig. 2.

### Exclusion criteria

The data of 0.25 ug/ml nafcillin on R10LB has been excluded from all studies due to the lack of statistically significant changes in presence of antibiotics (batch effect). The data and its result can be accessed in public repository or available upon request.

## Data Records

The reference genome *S*. *aureus* D712 was submitted in NCBI^15^. The growth-rate data is available on Figshare^21^, while BCP, HPLC, and Mass spectrometry data have been deposited on MassIVE repository^20^. Complete RNAseq pipeline can be found at Figshare^22^, Fastq files of each run has been deposited on NCBI- Sequence Read Archive^23^. More processed runs for RNAseq like TPM (batch corrected) and counts can be found on NCBI- Gene Expression Omnibus^24^. However, the overall summarized statistics of RNAseq is available on Figshare^25^.

## Technical Validation

### Bacterial cytological profiling

**Fig. 3:**
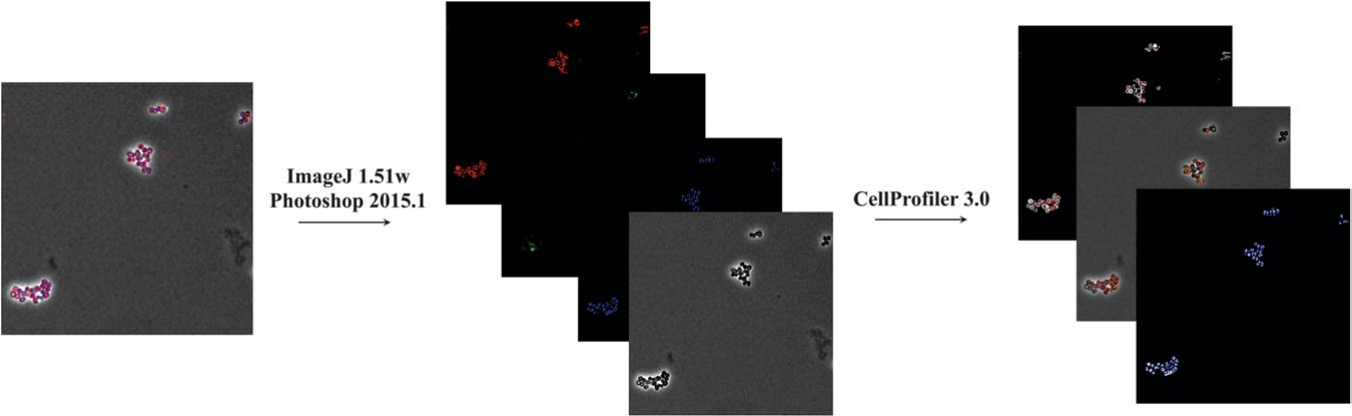
Depiction of image analysis pipeline for Bacterial cytological profiling of *Staphylococcus aureus* D712 in presence of nafcillin.

The technical validation of BCP was done through manual screening during the image segmentation process in CellProfiler. Accurate cell and object traces and measurements were verified manually for representative images. The cell outlines were matched with corresponding related structures e.g. DNA through “parent” tags. Finally, the output files for the cellular features were uploaded to MassIVE repository^20^. A representation of the image analysis pipeline for BCP data is provided in Fig. 3.

### Untargeted liquid chromatography mass spectrometry data acquisition

For each sample the base peak chromatogram (BPCs) and multiple extracted ion chromatograms (EICs) were compared to evaluate the reproducibility of global retention time and ion intensity. The reproducibility of retention time and peak intensity were obtained by comparing the BPCs of each experimental triplicate. While the EICs of the molecules were evaluated using retention time drift and peak area of <0.1 minutes and <15% correspondingly.

### RNA sequencing

The reference genome of D712 was sequenced using an Illumina Hiseq. 4000. Prior to assembly, the quality control steps were performed to remove unincorporated primers, adaptors, and detectable PCR primers. The genome was then assembled into 110 contigs using Spades v3.11 and annotated using Prokka v1.12. The sequencing reads shows the average Phred score of >32, which further corresponds to the base calling accuracy of 99.99%. The raw fastq files were uploaded at NCBI SRA web platform^23^. The alignment of reads with the reference genome gives the alignment score of 97.68%.

**Fig. 4:**
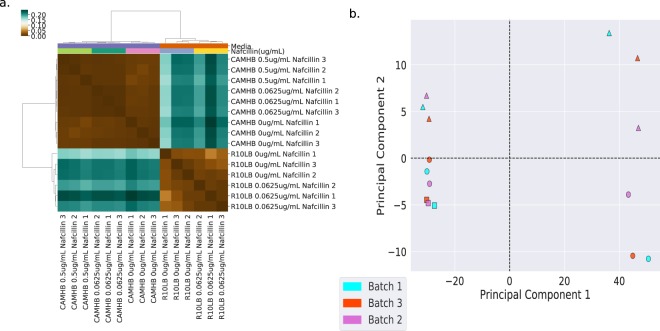
RNAseq results. (**a**) Clustering of reads TPM as per Spearman’s correlation coefficient. (**b**) PCA plot depicting the batch effect among samples.

As the number of samples were large so their processing was done in three different batches. Further, the batch effect of samples in each batch were corrected using ComBat module of SVA package in R, which resulted in high quality downstream data with Spearman’s correlation coefficient >0.95 among all biological replicates. The RNAseq results are shown in Fig. 4.

## Data Availability

The complete RNAseq pipeline used in analysis of RNAseq data is available on Figshare^22^. The script to remove batch effects from RNAseq data is also available of Figshare^26^.
